# Effect of the R92H and A379V genotypes of platelet-activating factor acetylhydrolase on its enzyme activity, oxidative stress and metabolic profile in Chinese women with polycystic ovary syndrome

**DOI:** 10.1186/s12944-017-0448-z

**Published:** 2017-03-20

**Authors:** Renjiao Zhang, Qi Song, Hongwei Liu, Huai Bai, Yujin Zhang, Qingqing Liu, Linbo Guan, Ping Fan

**Affiliations:** 1 Laboratory of Genetic Disease and Perinatal Medicine, Key Laboratory of Birth Defects and Related Diseases of Women and Children, Ministry of Education, West China Second University Hospital, Sichuan University, Chengdu, Sichuan 610041 People’s Republic of China; 2 Department of Obstetrics and Gynecology, West China Second University Hospital, Sichuan University, Chengdu, Sichuan 610041 People’s Republic of China

**Keywords:** PAF acetylhydrolase, Gene polymorphism, Enzyme activity, Oxidative stress, Polycystic ovary syndrome

## Abstract

**Background:**

The *G994T* polymorphism in platelet-activating factor acetylhydrolase (*PAF-AH*) gene is associated with the risk of polycystic ovary syndrome (PCOS). The aim of this study was to investigate the relationship between *R92H* and *A379V* variants of the *PAF-AH* gene and the risk of PCOS and to evaluate the effects of the genotypes on PAF-AH activities and clinical, metabolic and oxidative stress indexes in Chinese women.

**Methods:**

A total of 862 patients with PCOS based on the Rotterdam consensus criteria and 750 control women from a population of Chinese Han nationality in the Chengdu area were studied from 2006–2015. *PAF-AH* genotypes were determined by PCR and restriction fragment length polymorphism analysis. Plasma PAF-AH, high-density lipoprotein (HDL)-associated PAF-AH (H-PAF-AH) and apolipoprotein (apo) B-containing lipoprotein-associated PAF-AH (apoB-PAF-AH) activities were measured using the trichloroacetic acid precipitation procedure with PAF C-16 as a substrate. Circulating markers of oxidative stress, including serum total oxidant status, total antioxidant capacity, oxidative stress index and malondialdehyde levels, and clinical and metabolic parameters were also analyzed.

**Results:**

No significant differences were observed in the frequencies of *R92H* and *A379V* genotypes and alleles of the *PAF-AH* gene between PCOS and control groups (*P* > 0.05). Compared with patients with the *92RR* genotype, patients with *H* allele of *R92H* (*RH* + *HH* genotype) had significantly higher plasma PAF-AH and apoB-PAF-AH activities (*P* < 0.05) and tended to exhibit increased H-PAF-AH activity (*P* = 0.063) after adjusted for age and BMI. However, when serum LDL-C, HDL-C, TG and HOMA index were added as covariates, the comparisons no longer remained statistical significance (*P* > 0.05). There were no significant differences in clinical, hormonal, metabolic and circulating oxidative stress parameters and the frequencies of *PAF-AH G449T* genotype according to *PAF-AH R92H* or *A379V* genotyping in patients with PCOS and control women.

**Conclusions:**

There were no significant associations between *R92H* and *A379V* variants of *PAF-AH* gene and risk of PCOS in Chinese women. The increased plasma PAF-AH and apoB-PAF-AH activities in patients with *H* allele of *R92H* are related to the *R92* → *H* variation, changes in plasma lipoprotein levels, insulin resistance, aging, and gaining weight and thus may be involved in the pathogenesis of PCOS and the increased risks of future cardiovascular diseases.

## Background

Polycystic ovary syndrome (PCOS) is a heterogeneous female endocrine metabolic disorder affecting 5–15% of reproductive-age women [1, 2]. In addition to reproductive disorders, PCOS is often associated with long-term health risks, including insulin resistance, obesity, dyslipidemia, increased oxidative stress, chronic low-grade inflammation, endothelial dysfunction and vascular injury, elevated risks of metabolic syndrome, type 2 diabetes, and future cardiovascular diseases [2–9]. The etiology of PCOS remains obscure, but studies have suggested that PCOS has a complex, multifactorial etiology resulting from the interactions between genetic, environmental and intrauterine factors [10–12].

Plasma platelet-activating factor (PAF) acetylhydrolase (PAF-AH), also known as lipoprotein-associated phospholipase A2 (Lp-PLA2), is mainly bound to apolipoprotein (apo) B-containing lipoproteins, particularly low-density lipoprotein (LDL), and a small portion is also associated with high-density lipoprotein (HDL) [13, 14]. The abnormalities of plasma PAF-AH activity, mass and/or distribution in lipoproteins are associated with atherosclerosis and inflammatory diseases [14–16]. Decreased HDL-associated PAF-AH (H-PAF-AH) activities, increased apoB-containing lipoprotein-associated PAF-AH (apoB-PAF-AH) activities and/or the ratio of apoB-PAF-AH to total or H-PAF-AH activity are associated with cardiovascular diseases [14], type 2 diabetes [17], gestational diabetes mellitus [13], pre-eclampsia [18], and polycystic ovary syndrome [19, 20] and might be markers for chronic inflammation in these patients.

Several single-nucleotide polymorphisms (SNPs) in the exon region of the *PAF-AH* gene influence its activity or concentration. The *V279F (G994T)* SNP in exon 9 completely abolishes enzymatic activity in 279 *FF* homozygotes and results in a 50% decrease of catalytic activity in heterozygotes [19, 21]. The *V* allele of the *A379V* SNP in exon 11 results in a two-fold decrease in the affinity of PAF-AH for PAF [22]. The *H* allele of the *R92H* SNP in exon 4 is associated with a higher PAF-AH mass [16]. Several studies reported that the *PAF-AH R92H* and *A379V* variants were associated with increased risk of coronary heart disease (CHD), but the results were inconsistent [14, 15, 23, 24]. However, the evidence available to date strongly suggests that *PAF-AH* *G994* →* T* mutation is associated with increased risk of cardiovascular diseases in Japanese and Chinese cohorts [14, 24].

Previously Fan et al. demonstrated that the *T* allele of the *G994T* SNP in *PAF-AH* gene was one of the genetic determinants for PCOS in Chinese Han women [19]. Plasma PAF-AH hydrolyzes and inactivates PAF and PAF-like oxidized phospholipids, and is associated with circulating oxidative stress and inflammation status [14, 24]. However, to date, little information is available regarding the possible connection between the *PAF-AH* gene *A379V* and *R92H* SNPs and PCOS or oxidative stress. In the present study, we investigated the relationship between *R92H* and *A379V* variants of *PAF-AH* gene and the risk of PCOS and evaluated the effects of the genotypes on PAF-AH activities and clinical, metabolic and oxidative stress indexes in Chinese women.

## Methods

### Study subjects

This is a case-control study, which consists of 862 cases and 755 controls. The frequencies of *PAF-AH* genotype and allele are main variables. The sample sizes in the present study are reasonable and practicable according to a report by B-Rao [25].

Women with or without PCOS aged 17–40 years were recruited from 2006–2015 from the Outpatient Clinic of Reproductive Endocrinology, West China Second University Hospital.

Each woman with PCOS met diagnostic criteria for PCOS based on the revised 2003 Rotterdam ESHRE/ASRM consensus criteria [26]. Oligo- or anovulation (OA) was assessed as oligomenorrhea (i.e., fewer than eight cycles per year). Biochemical or clinical hyperandrogenism (HA) was assessed by total testosterone (TT) levels above the 95th percentile of the levels (2.60 nmol/l) detected in a group of normal menstruating women with normal cycles, hirsutism with a modified Ferriman–Gallwey (F-G) score of more than 6 and/or clinical presence of obvious acne [4, 27, 28]. Polycystic ovaries (PCOs) were confirmed if there were 12 or more follicles in each ovary measuring 2–9 mm in diameter and/or increased ovarian volume (>10 mL) by ultrasonic examination. The diagnosis of PCOS was based on a patient having two of these three findings for women aged 20–40 years or having all three findings for women aged < 20 years [2], with exclusion of other etiologies, such as androgen-secreting tumors, congenital adrenal hyperplasias, and Cushing syndrome. All of the control women had regular menstrual cycles (between 21 and 35 days), exhibited normal circulating androgen levels, the absence of hirsutism or obvious acne on physical examination, and normal ovarian morphology as determined by ultrasound.

None of the subjects had clinically evident chronic or acute diseases, such as infection, tumors, cardiovascular disease, thyroid dysfunction, endometriosis, hyperprolactinemia, hypogonadotropic hypogonadism or premature ovarian failure.

For association studies between *PAF-AH* genotypes and hormonal, metabolic and oxidative stress parameters, the subjects were excluded if they met one of the following criteria: [i] taking medication known to affect the metabolism of carbohydrates, lipids, or hormones within 3 months before the study; [ii] pregnant or in the luteal phase; and [iii] smokers.

Clinical and anthropometrical variables, including waist circumference, hip circumference, waist-to-hip ratio, body mass index (BMI, kg/m^2^), systolic and diastolic blood pressure (SBP and DBP), and the degree of hirsutism and acne were measured or assessed in all subjects. Ultrasound ovarian volume was also assessed using the formula [29]: 0.523 × length × width × thickness.

Blood samples were obtained in the morning after overnight fasting, placed on ice immediately and centrifuged at 1500 g for 15 min at 4 °C within 2 h. Plasma or serum aliquots were stored at −80 °C and blood cells were stored at 4 °C.

### Genotype analysis

Genomic DNA was isolated from peripheral blood leukocytes of subjects [28, 30]. The *PAF-AH R92H* and *A379V* genotypes were determined by PCR amplification and restriction analysis. For the *R92H* genotype, a 200-bp fragment was amplified using the following primers [31]: forward, 5’-ATGCAAAATAGCTGCTGGAA-3’ and reverse 5’-AATGTTGCCCATAAGCCAGT-3’. For the *A379V* genotype, a 99-bp fragment was amplified using the following primers [22]: forward, 5’-GGGAGACATAGATTCAACTG-3’ and reverse 5’-GGTCATGAAAAAAATAGTTT-3’. *R92H* and *A379V* PCR products were digested with BclI or PstI (MBI Fermentas, Vilnius, Lithuania), respectively, analyzed by electrophoresis on a 3.5% agarose gel and visualized by staining with Genecolour fluorescent dye. For the purpose of genotyping quality control, greater than 30% of DNA samples were genotyped again by the different operator.

### Measurements of plasma PAF-AH, H-PAF-AH and apoB-PAF-AH activities

HDL fractions were obtained by precipitating apoB-containing lipoproteins using 13% polyethylene glycol (PEG) 6000. The plasma PAF-AH and H-PAF-AH activities were measured via the trichloroacetic acid precipitation procedure as previously described [19, 32]. The intra- and inter-assay coefficients of variation for all measurements were less than 3% and 5%, respectively. The apoB-PAF-AH activity was obtained by subtracting the H-PAF-AH activity from plasma PAF-AH activity.

### Analysis of oxidative stress, hormonal and metabolic markers

Serum follicle stimulating hormone (FSH), luteinizing hormone (LH), TT, estradiol (E_2_), total cholesterol (TC), triglyceride (TG), HDL-cholesterol (HDL-C), LDL-cholesterol (LDL-C), apoA1, apoB, total oxidant status (TOS), total antioxidant capacity (T-AOC) and malondialdehyde (MDA) levels, plasma insulin and glucose concentrations as well as homeostatic model assessment of insulin resistance (HOMA index) and oxidative stress index (OSI) were measured or assessed as described before [4, 33, 34]. The intra- and inter-assay coefficients of variation for all measurements were less than 5% and 10%, respectively.

### Statistical analysis

Data were presented as the mean ± standard deviation (SD). Differences in variables were evaluated by the independent sample *t*-test between PCOS and control subjects. Variables with asymmetric distribution were evaluated by nonparametric tests (Mann-Whitney *U* test). *X* ^2^ analysis was used to test deviations of genotype distribution from Hardy-Weinberg equilibrium and to determine allele or genotype frequencies between patients and controls. Analysis of covariance was used to estimate the differences in metabolic and oxidative stress parameters and PAF-AH activities between two groups after correction for differences in covariates such as age, BMI and serum lipid levels. Pearson correlation was performed to define the correlations between PAF-AH activities and the other parameters in patients with PCOS. A *P*-value < 0.05 was considered to be statistically significant. All statistical analyses were performed using Statistical Program for Social Sciences (SPSS) 13.0 for Windows (Chicago, IL, USA).

## Results

### Clinical and biochemical characteristics of the study population

In accordance with the revised 2003 Rotterdam criteria, there were 358 cases (41.5%) with OA + HA + PCO, 276 cases (32%) with OA + PCO, 183 cases (21.2%) with OA + HA, and 45 cases (5.2%) with HA+ PCO in the PCOS group.

As shown in Table 1, BMI, waist circumference, waist-to-hip ratio, F-G score, DBP, and average ovarian volume were significantly increased, and age was significantly lower in the PCOS group compared with the control group.

**Table 1:** Clinical characteristics in PCOS patients and control women

	Controls (*n* = 750)	PCOS (*n* = 862)	*P*
Age (years)	28.16 ± 4.15	24.68 ± 3.90	0.000
BMI (kg/m^2^)	21.16 ± 2.95	22.79 ± 4.04	0.000
Waist circumference (cm)	73.60 ± 8.22	78.68 ± 11.12	0.000
Waist-to-hip ratio	0.82 ± 0.06	0.85 ± 0.07	0.000
F-G score	0.22 ± 0.72	1.68 ± 2.03	0.000
SBP (mmHg)	113.24 ± 11.46	114.20 ± 10.57	0.084
DBP (mmHg)	73.92 ± 8.97	75.62 ± 8.90	0.000
Ovarian volume (ml)	7.52 ± 2.87	9.89 ± 4.10	0.000

Values are presented as the mean ± SD

TT and LH levels, the ratio of LH to FSH, fasting insulin concentration, HOMA index, TG, TC, LDL-C, apoB and MDA levels, TOS, T-AOC, OSI, the ratio of apoB-PAF-AH to H-PAF-AH and the frequency of the *T* allele carriers (*GT + TT*) of *PAF-AH G994T* were significantly increased and FSH and HDL-C levels and H-PAF-AH activities were significantly reduced in the PCOS group compared with the control group after adjusted for age and BMI (Table 2). However, when serum LDL-C, HDL-C, TG and HOMA index were added as covariates, the comparisons of plasma PAF-AH, apoB-PAF-AH and H-PAF-AH activities as well as the ratio of apoB-PAF-AH to H-PAF-AH were statistically significant (*P* < 0.05).

**Table 2:** Hormonal, metabolic and oxidative stress parameters and PAF-AH activities in PCOS patients and control women

	Controls (*n* = 501)	PCOS (*n* = 565)	*P*	*P* ^*a*^
Age (years)	27.93 ± 4.20	24.69 ± 3.90	0.000	
BMI (kg/m^2^)	21.07 ± 2.89	23.08 ± 4.24	0.000	
Hormonal levels				
E_2_ (pmol/L)	331.82 ± 350.14	286.60 ± 272.76	0.027	0.587
TT (nmol/L)	1.53 ± 0.56	2.43 ± 0.76	0.000	0.000
LH (IU/L)	8.63 ± 10.94	13.93 ± 10.97	0.000	0.000
FSH (IU/L)	6.67 ± 2.97	5.99 ± 2.25	0.000	0.018
LH/FSH	1.30 ± 1.31	2.36 ± 1.27	0.000	0.000
Metabolic profile and oxidative stress parameters				
Fasting Ins (pmol/L)	66.12 ± 35.49	105.43 ± 72.16	0.000	0.000
Fasting Glu (mmol/L)	5.30 ± 0.74	5.37 ± 0.78	0.108	0.473
HOMA-IR	2.32 ± 1.85	3.77 ± 3.13	0.000	0.001
TG (mmol/L)	1.04 ± 0.90	1.43 ± 1.37	0.000	0.000
TC (mmol/L)	4.24 ± 0.70	4.41 ± 0.81	0.000	0.000
HDL-C (mmol/L)	1.51 ± 0.32	1.38 ± 0.35	0.000	0.008
LDL-C (mmol/L)	2.34 ± 0.61	2.55 ± 0.76	0.000	0.000
ApoA1 (g/L)	1.45 ± 0.21	1.41 ± 0.21	0.005	0.772
ApoB (g/L)	0.75 ± 0.17	0.82 ± 0.20	0.000	0.000
TOS (nmol H_2_O_2_ Equiv./mL)	11.20 ± 5.31	14.96 ± 10.81	0.000	0.000
T-AOC (U/ml/min)	14.54 ± 2.67	15.81 ± 3.07	0.000	0.000
OSI	0.80 ± 0.40	1.00 ± 0.80	0.000	0.000
MDA (nmol/ml)	3.65 ± 1.08	4.36 ± 1.13	0.000	0.000
PAF-AH activities and frequencies of *PAF-AH G449T* genotype^a^				
Plasma PAF-AH (nmol/min/ml)	48.34 ± 10.81	47.44 ± 12.94	0.371	0.741
H-PAF-AH (nmol/min/ml)	5.11 ± 1.48	4.66 ± 1.91	0.002	0.038
ApoB-PAF-AH (nmol/min/ml)	43.25 ± 9.96	42.77 ± 11.89	0.625	0.976
ApoB-PAF-AH/H-PAF-AH	8.88 ± 2.47	10.04 ± 3.89	0.000	0.000
*PAF-AH G449T*, n (%)				
*GG*	255 (92.7%)	243 (86.5%)		
*GT + TT*	20 + 0 (7.3%)	34 + 4 (13.5%)	0.020	

Values are presented as the mean ± SD

*ApoA1* apolipoprotein A1, *apoB* apolipoprotein B, *TOS* total oxidant status, *T-AOC* total antioxidant capacity, *OSI* oxidative stress index, *PAF-AH* platelet activating factor acetylhydrolase, *H-PAF-AH* HDL-associated PAF-AH, *ApoB-PAF-AH* apolipoprotein B-containing lipoprotein-associated PAF-AH

*P* ^*a*^ All comparisons of parameters were corrected for differences in age and BMI between the two groups

^a^Controls (*n* = 275), PCOS (*n* = 281)

### Distribution of *PAF-AH R92H* and *A379V* genotypes and alleles

Genotypic distributions of *PAF-AH R92H* and *A379V* were in Hardy-Weinberg equilibrium in the PCOS and control groups. No significant differences were observed in the frequencies of *PAF-AH R92H* and *A379V* genotypes and alleles between PCOS and control groups (*P* > 0.05, Table 3).

**Table 3:** Frequencies of *PAF-AH* genotype and allele in PCOS patients compared with control women

		Controls (*n* = 750)	PCOS (*n* = 862)	*X* ^*2*^	*P*
Genotype					
92	RR	486 (64.8%)	535 (62.1%)		
	RH	238 (31.7%)	294 (34.1%)		
	HH	26 (3.5%)	33 (3.8%)	1.302	0.522
379	AA	564 (75.2%)	647 (75.1%)		
	AV	171 (22.8%)	202 (23.4%)		
	VV	15 (2.0%)	13 (1.5%)	0.629	0.700
Allele frequency					
92	R	0.807	0.791		
	H	0.193	0.209	1.195	0.274
379	A	0.866	0.868		
	V	0.134	0.132	0.021	0.884

Data of genotype are presented as number (%) of patients or controls

### Effects of *PAF-AH R92H* and *A379V* genetic variants on clinical, hormonal, metabolic and oxidative stress parameters and PAF-AH activities

We further analyzed effects of *PAF-AH R92H* and *A379V* genetic variants on oxidative stress parameters and PAF-AH activities as well as clinical, hormonal, metabolic parameters in PCOS patients and control women. Because the sample sizes of the *92HH* or *379VV* homozygotes were too small, we combined them into heterozygous subgroups.

As shown in Table 4, compared with patients with *92 RR* genotype, patients with *H* allele of *R92H* (*RH* + *HH* genotype) had significantly higher plasma PAF-AH (*P* = 0.034) and apoB-PAF-AH activities (*P* = 0.045) and tended to exhibit increased H-PAF-AH activity (*P* = 0.063) after adjusted for age and BMI. Similar alterations were also observed in the control group, but the values did not reach statistical significance (*P* > 0.05). In addition, when serum LDL-C, HDL-C, TG and HOMA index were added as covariates, plasma PAF-AH, apoB-PAF-AH and H-PAF-AH activities no longer remained statistical significance between patients with *92 RR* genotype and patients with *H* allele (*P* = 0.054, 0.243 and 0.183, respectively). No significant differences in oxidative stress indexes and the frequencies of *PAF-AH G449T* genotype (Table 4) and clinical, hormonal, metabolic parameters (data not shown) according to *PAF-AH R92H* genotyping were detected in PCOS patients and control women (*P* > 0.05).

**Table 4:** Oxidative stress parameters and PAF-AH activities according to *PAF-AH R92H* genotype in PCOS patients and control women

	Controls		PCOS	
	RR (*n* = 335)	RH + HH (*n* = 151 + 15)	RR (*n* = 357)	RH + HH (*n* = 186 + 22)
Age (yr)	28.10 ± 4.23	27.58 ± 4.12	24.98 ± 3.29	24.20 ± 3.81^a^
BMI (kg/m^2^)	21.14 ± 3.05	20.92 ± 2.53	23.09 ± 4.33	23.07 ± 4.10
Oxidative stress parameters				
TOS (nmol H_2_O_2_ Equiv./mL)	11.27 ± 5.49	10.99 ± 4.92	15.42 ± 11.83	14.17 ± 8.88
T-AOC (U/ml/min)	14.38 ± 2.58	14.85 ± 2.84	15.84 ± 3.21	15.80 ± 2.83
OSI	0.81 ± 0.42	0.76 ± 0.37	1.02 ± 0.84	0.95 ± 0.72
MDA (nmol/ml)	3.67 ± 1.10	3.61 ± 1.04	4.31 ± 1.25	4.42 ± 1.14
PAF-AH activities and frequencies of *PAF-AH G449T* genotype*				
Plasma PAF-AH (nmol/min/ml)	48.02 ± 11.14	48.90 ± 10.26	46.30 ± 13.32	49.37 ± 12.08^a^
H-PAF-AH (nmol/min/ml)	5.04 ± 1.41	5.25 ± 1.59	4.51 ± 1.75	4.92 ± 2.14
ApoB-PAF-AH (nmol/min/ml)	42.98 ± 10.31	43.64 ± 9.37	41.79 ± 12.23	44.45 ± 11.14^a^
ApoB-PAF-AH/H-PAF-AH	8.94 ± 2.60	8.77 ± 2.25	10.15 ± 4.37	9.85 ± 2.95
*PAF-AH G449T*, n (%)				
*GG*	158 (90.8%)	97 (96.0%)	150 (84.7%)	93 (89.4%)
*GT + TT*	16 + 0 (9.2%)	4 + 0 (4.0%)	23 + 4 (15.3%)	11 + 0 (10.6%)

Values are presented as the mean ± SD

*TOS* total oxidant status, *T-AOC* total antioxidant capacity, *OSI* oxidative stress index, *PAF-AH* platelet activating factor acetylhydrolase, *H-PAF-AH* HDL-associated PAF-AH, *ApoB-PAF-AH* apolipoprotein B-containing lipoprotein-associated PAF-AH

Comparisons of oxidative stress parameters and PAF-AH activities were corrected for differences in age and BMI between the two groups

^a^ *P* < 0.05, compared with *RR* genotype subgroup in PCOS patient

^*^Controls: RR (*n* = 174), RH + HH (*n* = 88 + 13); PCOS: RR (*n* = 177), RH + HH (*n* = 98 + 6)

No significant differences in oxidative stress indexes, the frequencies of *PAF-AH G449T* genotype and PAF-AH activities (Table 5) as well as other clinical, hormonal, and metabolic parameters (data not shown) according to *PAF-AH A379V* genotypes were detected in PCOS patients and control women (*P* > 0.05).

**Table 5:** Oxidative stress parameters and PAF-AH activities according to *PAF-AH A379V* genotype in PCOS patients and control women

	Controls		PCOS	
	AA (*n* = 371)	AV + VV (*n* = 121 + 9)	AA (*n* = 432)	AV + VV (*n* = 125 + 8)
Age (yr)	27.85 ± 4.33	28.15 ± 3.80	24.79 ± 3.95	24.39 ± 3.70
BMI (kg/m^2^)	20.98 ± 3.00	21.33 ± 2.53	23.21 ± 4.41	22.66 ± 3.62
Oxidative stress parameters				
TOS (nmol H_2_O_2_ Equiv./mL)	11.06 ± 5.13	11.51 ± 5.81	14.95 ± 11.25	15.00 ± 9.43
T-AOC (U/ml/min)	14.58 ± 2.62	14.39 ± 2.86	15.82 ± 3.12	15.86 ± 2.92
OSI	0.78 ± 0.39	0.83 ± 0.44	1.00 ± 0.84	0.99 ± 0.65
MDA (nmol/ml)	3.64 ± 1.05	3.69 ± 1.15	4.38 ± 1.31	4.27 ± 1.31
PAF-AH activities and frequencies of *PAF-AH G449T* genotype^a^				
Plasma PAF-AH (nmol/min/ml)	48.22 ± 10.85	48.68 ± 10.79	47.37 ± 13.78	47.70 ± 9.09
H-PAF-AH (nmol/min/ml)	5.17 ± 1.57	4.96 ± 1.21	4.67 ± 2.03	4.66 ± 1.40
ApoB-PAF-AH (nmol/min/ml)	43.04 ± 9.92	43.72 ± 10.12	42.70 ± 12.63	43.04 ± 8.53
ApoB-PAF-AH/H-PAF-AH	8.79 ± 2.52	9.13 ± 2.34	9.97 ± 3.07	10.31 ± 6.10
*PAF-AH G449T*, n (%)				
*GG*	185 (92.0%)	70 (94.6%)	190 (85.2%)	53 (91.4%)
*GT + TT*	16 + 0 (8.0%)	4 + 0 (5.4%)	29 + 4 (14.8%)	5 + 0 (8.6%)

Values are presented as the mean ± SD

*TOS* total oxidant status, *T-AOC* total antioxidant capacity, *OSI* oxidative stress index, *PAF-AH* platelet activating factor acetylhydrolase, *H-PAF-AH* HDL-associated PAF-AH, *ApoB-PAF-AH* apolipoprotein B-containing lipoprotein-associated PAF-AH

Comparisons of oxidative stress parameters and PAF-AH activities were corrected for differences in age and BMI between the two groups

^a^Controls: AA (*n* = 201), AV + VV (*n* = 70 + 4); PCOS: AA (*n* = 223), AV + VV (*n* = 55 + 3)

## Discussion

In the present study, we show that *PAF-AH R92H* and *A379V* genetic polymorphisms are not associated with the risk of PCOS in Chinese women. However, we found that plasma PAF-AH and apoB-PAF-AH activities in patients carrying the minor allele of *R92H* polymorphism of *PAF-AH* gene are increased compared with those homozygous for the wild-type allele, suggesting that the *R92H* variant may be associated with increased plasma and lipoprotein-associated PAF-AH activities in these patients. In addition, our results revealing no significant differences in TOS, OSI and MDA levels according to *R92H* or *A379V* genotypes in PCOS and control groups suggests that these genetic variants that modestly or minimally impact enzymatic activity are unlikely to significantly affect circulating oxidative stress levels.

Plasma PAF-AH specifically hydrolyzes and inactivates PAF and PAF-like oxidized phospholipids that are potent pro-inflammatory mediators and thus plays an anti-inflammatory role [14, 24]. Genetic studies in humans harboring an inactivating mutation (*PAF-AH* *G994* → * T* mutation) indicate that loss of PAF-AH activity is a risk factor for inflammatory and cardiovascular diseases in Japanese cohorts [14, 24]. Consistently, overexpression of PAF-AH has anti-inflammatory and anti-atherogenic properties in animal models [14, 24, 35]. However, several clinical studies have demonstrated that plasma PAF-AH activity and mass are strongly associated with atherogenic lipids and risk of cardiovascular disease [14, 16, 24, 36]. The hydrolysis products of this enzyme, lysophosphatidylcholine (lyso-PC) and oxidatively modified nonesterified fatty acids, promote the pathogenesis of atherosclerosis [14, 24, 35]. Thus, controversy remains regarding whether plasma PAF-AH exerts a pro- or anti-inflammatory action. Given that H-PAF-AH, one of antioxidant enzymes of HDL, plays anti-inflammatory role [14, 19] and apoB-PAF-AH is associated with inflammation [13, 14, 24], it has recently been suggested that the relative distribution of the enzyme between apoB-containing lipoproteins and HDL determines its pro- or anti-inflammatory actions. Previous studies indicated that H-PAF-AH activity was decreased and the ratio of LDL-associated PAF-AH to H-PAF-AH activities and MDA levels were increased in women with PCOS, and the *PAF-AH* *G994* → * T* gene mutation is a risk factor for PCOS [19, 20, 32]. Consistent with previous studies, this study further determined that patients with PCOS had significantly higher TOS, OSI, and the ratio of apoB-PAF-AH to H-PAF-AH activities compared with the control women, suggesting that increased circulating absolute (TOS) and relative (OSI) oxidative stress levels and chronic inflammation existed in these patients.

Several studies have demonstrated that the *PAF-AH 92R* → *H* genetic variant was associated with an increased PAF-AH mass [15, 16, 23] and was a risk factor of CHD [23, 37]. However, in meta-analyses, the *PAF-AH R92H* genetic polymorphism was not significantly associated with the risk of CHD [15]. In the present study, we showed that this genetic polymorphism was not associated with the risk of PCOS in Chinese women. However, the *PAF-AH 92R* → *H* variant was significantly associated with increased plasma and lipoprotein-associated PAF-AH activities in PCOS patients after adjusted for age and BMI, but when LDL-C, HDL-C, TG, and HOMA-IR were added as covariates, the statistical differences were no longer maintained. Grallert et al [15] indicated that PAF-AH activity was strongly associated with genetic variants involved in lipid metabolism. A previous study also demonstrated that the ratio of L-PAF-AH to H-PAF-AH activities was positively correlated with LDL-C, TG, HOMA-IR, fasting plasma insulin and glucose concentrations, and negatively correlated with HDL-C levels in patients with PCOS [20]. Similarly, the present study indicated that plasma PAF-AH and apoB-PAF-AH activities were positively correlated with LDL-C, apoB, TC, TG, age, SBP, waist circumference, waist-to-hip ratio, and HOMA-IR (*P* < 0.05) and negatively correlated with HDL-C levels (*P* < 0.05) in the patients according to the correlation analysis (data not shown). In addition, evidence suggests that PAF, oxidized LDL, and oxidized phospholipids upregulate expression of PAF-AH in vivo [24]. These results suggest that multiple factors including the *92R* → *H* variant, changes in plasma lipoprotein levels, increased oxidative stress or/and chronic inflammation, insulin resistance, aging, and gaining weight may contribute to increased plasma and lipoprotein-associated PAF-AH activities according to *PAF-AH R92H* genotypes in PCOS patients.

The *PAF-AH 379A* → *V* variant results in a two-fold decrease in the affinity of PAF-AH for its substrates [22], thus prolonging the pro-inflammatory actions of PAF and PAF-like oxidized phospholipids in plasma. However, the effect of this variant on PAF-AH activity or disease is conflicting. The *379A* → *V* variant is associated with increased [15, 31, 36] or decreased [38] activity and increased [38, 39], decreased [40, 41] or no affect [15, 36] on the risk of cardiovascular diseases. Our study determined that the *A379V* SNP was not significantly associated with the risk of PCOS and PAF-AH activities in Chinese women.

In particular, the allele frequencies for *PAF-AH* genetic variants differ among ethnic groups [14, 24]. For example, the *G994T* SNP is common in Japanese and Taiwan populations and has been reported in Korean, Chinese, Turkey populations; however, this SNP is rarely observed in Europeans [24]. In the present study, the frequency of the *T* allele carriers (*GT* + *TT*) of *PAF-AH G994T* was significantly increased in the PCOS group compared with the control group (*P* = 0.02, Table 2). This may be an important reason why patients with PCOS have higher TC, TG and LDL-C levels, but have relatively lower plasma PAF-AH and apoB-PAF-AH activities. Because The *G994 → T* variation completely abolishes enzymatic activity in *TT* homozygotes and results in a 50% decrease of catalytic activity in heterozygotes [19, 21]. In addition, environmental factors, such as increased oxidative stress, inflammation status, a high-fat diet, and lipoprotein levels may also affect PAF-AH expression or the distribution of this enzyme in lipoproteins [16, 19, 20, 24]. Therefore, it is possible that the relationships between *PAF-AH* genetic polymorphisms and PAF-AH activities or diseases may differ among different populations.

Regarding limitations of the present study, given the low frequency of homozygosity of minor alleles, *92HH* and *379VV*, we could not analyze them in the form of subgroups, and a larger number of subjects are needed to properly evaluate dose dependent genotype characteristics. Second, due to insufficient sample, we did not assess plasma and lipoprotein-associated PAF-AH activities in some subjects, which might influence the power of these parameters or result in the absence of statistical significances. Further study to increase the sample size for PAF-AH activity determination may help improve statistical power of these parameters between the two different genotype subgroups.

## Conclusion

The present study demonstrates that *PAF-AH R92H* and *A379V* genetic polymorphisms are not associated with the risk of PCOS and the circulating absolute and relative oxidative stress levels in Chinese women. The increased plasma PAF-AH and apoB-PAF-AH activities in patients with *H* allele of *R92H* are related to multiple factors including the *R92* → *H* variation, changes in plasma lipoprotein levels, insulin resistance, aging, and gaining weight and thus might be involved in the pathogenesis of PCOS and the increased risks of future cardiovascular diseases in the patients.

## Abbreviations

apoA1: apolipoprotein A1
apoB: apolipoprotein B
apoB-PAF-AH: apoB-containing lipoprotein-associated PAF-AH
BMI: body mass index
DBP: diastolic blood pressure
E_2_: estradiol
F-G score: Ferriman–Gallwey score
FSH: follicle stimulating hormone
Glu: glucose
HA: hyperandrogenism
HDL: high-density lipoprotein
HDL-C: HDL cholesterol
HOMA index: homeostatic model assessment of insulin resistance
H-PAF-AH: HDL-associated PAF-AH
Ins: insulin
LDL: low-density lipoprotein
LDL-C: LDL cholesterol
LH: luteinizing hormone
MDA: malondialdehyde
OA: Oligo- or anovulation
OSI: oxidative stress index
PAF-AH: platelet activating factor acetylhydrolase
PCOS: polycystic ovary syndrome
SBP: systolic blood pressure
SNP: single-nucleotide polymorphism
T-AOC: total antioxidant capacity
TC: total cholesterol
TG: triglycerides
TOS: total oxidant status
TT: total testosterone
